# Do methodological limitations and confounding factors explain the apparent acceleration of cognitive decline in older adults taking omega-3 fatty acid supplements?

**DOI:** 10.1016/j.tjpad.2026.100661

**Published:** 2026-08-18

**Authors:** Simon C. Dyall, Mélanie Plourde

**Affiliations:** a School of Life and Health Sciences, University of Roehampton, London, UK; b Research Center on Aging, CIUSSS de l'Estrie - CHUS, Sherbrooke, QC, Canada; c Department of Medicine, Université de Sherbrooke, Sherbrooke, QC, Canada

**Keywords:** Alzheimer's disease, Omega-3 fatty acids, Cognitive decline, Amyloid-β, Tau, Synaptic dysfunction

## Abstract

Not applicable

Dear Editor,

We read with interest the recent report by Liao and colleagues [1]. These findings are in direct opposition to Wei *et al*., who on the same ADNI cohort reported that omega-3 supplementation was associated with a 64% reduced risk of Alzheimer’s Disease (AD) (HR: 0.36, 95% CI: 0.18, 0.72; P = 0.004) [2]. In this letter, we draw attention to methodological and interpretive concerns that, in our view, were not adequately addressed in the article by Liao et al.*,.* and may explain these discordant observations.

Omega-3 users and non-users were matched for age, sex composition, frequencies of APOE ε4 carriers, diagnosis, education, ADAS-Cog13 scores, FDG-SUVR, stroke, insomnia, depression, anxiety, and Parkinson’s disease. However, what is not discussed is that for many of these characteristics there were very high numbers of missing values, (Table 1).

**Table 1:** Number and percentage of participants with missing demographic characteristics.

Characteristic	Omega-3 non-users (546)*	Omega-3 users (273)*
Education	371 (68%)	104 (38%)
ADAS-Cog13	227 (42%)	167 (61%)
FDG SUVR	148 (27%)	82 (30%)
Stroke	275 (50%)	217 (79%)
Parkinson	226 (41%)	166 (61%)
Insomnia	497 (91%)	222 (81%)
Anxiety	275 (50%)	217 (79%)
Depression	275 (50%)	217 (79%)

⁎ Total number participants per group Abbreviations: ADASCog13, Alzheimer’s Disease Assessment Scale-Cognitive subscale 13; FDG, fluorodeoxyglucose; SUVR, standardized uptake value ratio.

Of note, for insomnia 91% and 81% of omega-3 non-users and users, respectively, had missing values, which is a significant risk factor for dementia. This missing data limits the ability to match groups appropriately, and by extension this information cannot be included in modelling. Indeed, only four characteristics were included (age, sex, APOE ε4 status, and diagnosis), thereby limiting the potential to control for many important dementia risk factors.

Wei et al., analysed 1135 participants without dementia and included in their model age, sex, education, cognitive status (MCI vs normal cognition), APOE ε4 status, medical history (hyperlipidemia, hypertension, diabetes, stroke history, insomnia, depression, anxiety, smoking status, coronary artery disease), alcohol intake, multivitamin, vitamin B12, and folate supplementation, anti-hypertensive drugs, and anti-diabetic drugs, and BMI [2]. Many of these are particularly important in omega-3 PUFA research, and lack of their consideration is an important factor in the inconsistent findings of clinical trials with omega-3 fatty acids [3].

The authors address the potential for reverse causality by comparing longitudinal cognitive trajectories; however, this approach only incorporates cognitive changes, and not wider potential drivers of intake, such as cardiovascular health, depression, and anxiety, which are all risk factors for AD. Furthermore, approximately 40% of participants were APOE4 carriers, the strongest genetic risk factor for late-onset AD. This proportion is substantially higher than that observed in most population-based cohorts [4], raising concerns about representativeness. APOE4 carriers are more vulnerable to accelerated cognitive decline and metabolic dysfunction and due to the very limited range of characterises included in the modelling, the issues of reverse causality, selection bias, and wider generalizability of the findings remain.

The categorisation of omega-3 supplementation was based on self-reported intake at a single timepoint, and provides no indication of dose, duration, compliance, or type of omega-3 fatty acid. The authors suggest that the exposure definition was validated by analysis of serum omega-3 levels. However, no information is provided about which lipid fraction was analysed nor whether the participants were fasted (as values can be highly influenced by recent dietary intake [5]), or the timing of the sampling or the number of participants included in the analysis. This all limits the confidence that can be apportioned to this categorisation.

There are also important inconsistencies in the alignment of cognitive outcomes. The correlation plots indicate that individuals receiving omega-3 supplementation exhibited extremely low MMSE scores (<10), very high ADAS-Cog scores (>60), and markedly elevated CDR scores (>15). While MMSE and ADAS-Cog values in these ranges are consistent with severe dementia, corresponding CDR scores would typically fall within a moderate stage. This discordance is clinically implausible and suggests potential issues with score scaling, data harmonization, or analytical processing. Such internal inconsistency substantially weakens confidence in the reported associations.

Importantly, attributing such profound cognitive decline to omega-3 supplementation alone is highly unlikely. This interpretation is further undermined by the absence of concordant changes in established biomarkers of AD pathology. Notably, the authors report no association between omega-3 supplementation and longitudinal changes in amyloid-β deposition, tau aggregation, or grey matter atrophy. Given the well-established relationship between cognitive decline and these neuropathological processes, it is difficult to reconcile severe cognitive deterioration without parallel biological progression. This discrepancy strongly suggests the presence of unaccounted confounding factors or biases.

The authors also report increased FDG hypometabolism in omega-3 supplemented participants and interpret this as evidence of synaptic injury, potentially driven by oxidative stress related to DHA peroxidation. However, this mechanistic explanation is not supported by the data presented. No measures of brain DHA levels, lipid peroxidation products, neuronal integrity, or mitochondrial function were included. In the absence of such evidence, the proposed link between DHA peroxidation and reduced glucose metabolism remains speculative. Therefore, this interpretation appears overstated and insufficiently grounded in the empirical findings of the study.

Given these widespread limitations and the highly speculative nature of the drivers of adverse effects by the authors, we consider a much more cautious interpretation of these findings is warranted.

Sincerely,

## CRediT authorship contribution statement

**Simon C. Dyall:** Conceptualization, Investigation, Writing – original draft, Writing – review & editing. **Mélanie Plourde:** Conceptualization, Investigation, Writing – original draft, Writing – review & editing.

## Declaration of competing interest

The authors declare that they have no known competing financial interests or personal relationships that could have appeared to influence the work reported in this paper.
